# The increased of MMP-9 and MMP-2 with the decreased of TIMP-1 on the uterosacral ligament after childbirth

**DOI:** 10.11604/pamj.2018.30.283.9905

**Published:** 2018-08-23

**Authors:** Rahajeng Rahajeng

**Affiliations:** 1 Division of Urogynecology, Department of Obstetrics and Gynecology, Medical Faculty, Brawijaya University/Saiful Anwar Hospital of Malang, Indonesia

**Keywords:** MMP-9, MMP-2, TIMP-1, primigravida

## Abstract

**Introduction:**

Pelvic floor dysfunction is one of the causes of morbidity that have very active role in the degradation of collagen and elastin through an intermediary changes in matrix metalloproteinases (MMP) and its regulation. This research to evaluate level of MMP-9, MMP-2 and TIMP-1 expression on uterosacral ligament in women after vaginal delivery.

**Methods:**

This research was conducted by collecting samples in consecutive sampling of biopsy uterosacral ligament from primigravida who underwent cesarean section because of obstructive labor at stage 1 and 2. As control, biopsy samples were taken from uterosacral ligament of primigravida a term who have not entered the labor phase and undergoing elective caesarean section surgery. The method in this study was cross sectional. The examination of MMP-9, MMP-2, and TIMP-1 expression by immunohistochemistry based on comparison of the sample and the control differences.

**Results:**

There are differences in the levels of Matrix Metalloproteinase-9, Matrix Metalloproteinase-2, and TIMP-1 in the uterosacral ligament of primigravida women in labor compared with women who are never in labor.

**Conclusion:**

There are increased levels of Matrix Metalloproteinase-9 and Matrix Metalloproteinase-2 with the decreased of TIMP-1 in the uterosacral ligament of primigravida women who underwent cesarean section because of obstructive labor at stage 1 and 2.

## Introduction

Pelvic floor dysfunction is one of the causes of morbidity that can degrade the quality of high prevalence life, although the main cause is unknown [1]. Pelvic organ prolapse (POP) or urogenital prolapse, is a decline in the pelvic organs that cause protrusion of the vagina, uterus or both. This situation can damage the walls of the anterior, posterior vagina, and uterus or vagina peak, which is generally seen as a combination of several circumstances above [2]. It is estimated that 11% of all women have underwent at least one surgical procedure POP [3]. In 1997, more than 225,000 patients who have pelvic organ prolapse surgery in the USA (22.7/10000 women), with estimates of more than 1 billion financing US$. POP is an indication of the hysterectomy in postmenopausal women and about 15-18% of all procedures in all age groups. This condition rarely causes severe morbidity or mortality; but quite a symptomatic lower genital, urinary and gastro intestinal tract that can affect women daily activities and quality of women life [2]. Although the mechanism of pelvic organ prolapse in women and the factors that led to the failure of the repair operation is not yet fully understood, there are some evidences to suggest that abnormalities in the structure of connective tissue is a predisposing factor [1]. In spite of the high incidence of POP, only a little cases is known underlying pathophysiological basis. Age, occupation, weight/body mass index (BMI), parity, type of delivery, vaginal delivery using a vacuum or forceps, the child´s weight biggest born, surgical history, history of medical ailments, menopausal status and the use of hormone replacement therapy are risk factors which often associated with the incidence of POP [4]. The process of pregnancy and childbirth are closely related to the incidence of pelvic organ prolapse. This is because the fetus through the birth canal during delivery causes maximum distensibility in the pelvic floor, causing an injury to the pelvic floor. In addition, tissue trauma including pelvic floor can be followed by imperfect healing of laceration postpartum so it will cause permanent damage to the network. This damage causes the failure of the pelvic floor serves as a support and thus increases the risk of pelvic organ prolapse [5].

The strength of the pelvic organs rely on muscle and loose connective tissue that binds to bone to form a proponent of the pelvic floor that is beneath the abdominal cavity [6]. Pelvic organs are supported by the pelvic floor muscles and fascia attachments on endopelvic [2]. The knowledge of the normal pelvic organ support is required to understand the pathophysiology of pelvic organ prolapse. The strength of the pelvic floor ligaments and fascia that vary among individuals is one of the important factors of prolapse incidence. One important part of the pelvic supporting system is the pelvic uterosacral ligament. This ligament provides major support to the cervix and upper vagina wall [7]. In vitro studies indicate that the cervical portion of the uterosacral ligament supporting over 17 kg weight before decreasing function [1]. The key element of this network is the quantity of stability, ultra structure, and organization of extracellular matrix proteins such as collagen, fibronectin, and elastin, includes receptors such as integrins [8]. The degradation process depends on the activity of matrix metalloproteinases (MMP) and its regulation, activation or sequestration growth factor, growth factor binding protein, cell surface receptors, and adhesion between cells. MMP is a protein that is structurally interconnected that degrade the extracellular matrix and basement of membrane components. The degradation process of collagen and elastin is mainly depend on the activity of regulatory MMP [9]. There is an increased degradation of elastin and collagen postpartum which may contribute to the pathogenesis of pelvic prolapse which is common in postpartum. In vivo studies that assess the expression of MMP and TIMP in delivery and post-delivery has not been reported. The changes in collagen and elastin through an intermediary changes in MMP-9 and TIMP-1 which have very active role in the degradation of collagen and elastin are not yet fully understood. In pathophysiology, the delivery process to change the molecular and biochemical basis mainly on the pelvic sacrouterine ligament, the walls of the vagina and levatorani, in causing prolapse is still studied. The changes in MMP-9 and TIMP-1 due to the strain that has been demonstrated in mice after birth, stimulates us to investigate further about the same effect as a result of the strain that occurs in pregnant women in labor compared with women who are never in labor, in this case the strain which on the uterosacral ligaments on human.

## Methods

**Sample preparation:** This study used analytic observational design with cross sectional types. Sampling was carried out in the delivery room departement-Obstetrics and Gynecology of dr. Saiful Anwar Hospital Malang, Ngudiwaluyo Hospital Wlingi, and dr. Iskak Tulungagung Hospital, Indonesia. The examinations were conducted at the Laboratory of Biomedical Faculty of Medicine, Brawijaya University. Population of the study objects was healthy women aged 20 to 40 years and agreed to be involved in research. The samples were taken at the uterosacral ligament from the population according to the inclusion criterias and variables that have been de-termined. Selection of the samples was done by using purposive sampling technique of inclusion and exclusion criteria that have been determined. The sample size used for each sample group was 11 people in minimum. Sampling of uterosacral liga-ment was taken at the operating room during cesarean section. Uterine stretched toward caudal to expose the uterosacral ligament. Uterosacral ligament held by using tweezers anatomical then using Metzenbaum scissors performed the sampling size of ±3mm - 5cm. Samples were inserted into the formalin tube. The tube was labeled accordance with the specimen. Storage samples were at temperature 20-25^o^C. Then prepared sample for immunohistochemical examination.

**Protein expression analysis:** Calculation procedure expression of MMP-9, MMP-2, and TIMP-1: Examination conducted on each slide using a light microscope at 400x magnification field and examined on 10 each object field of view. Imaging results were in the form of files(.jpeg) uploaded then processed through applications JPEG2000 Immuno Ratio virtual microscope slide, online application from the Institute of Biomedical Technology Tampere Finland. This application calculates the percentage of nuclear area which was smeared positive (labeling index) by using algorithms to separate components of color deconvulation outward appearance. Results obtained from the output in the form of presentation DAB smeared area of the total area of the nucleus. To ensure the representation and reduce errors in the results, the observations were necessary to examine approximately 10 field of view with 400x magnification.

**Statistical analysis:** Independent sample t test is a statistical analysis method to prove the hypothesis of the study that has been submitted. It will be used to test the statistical parametric to compare the mean of the levels of MMP-9, MMP-2 and TIMP-1 in the sacrouterine ligament among inpartu pregnant women and non-inpartu pregnant women. The criteria for the decision was by looking at the value of p-value, if it was greater than the significance level α=0.05 then the conclusion will be there was no significantly increase/decrease in inpartu pregnant women and if it is smaller than the significance level α=0.05, the conclusion will be there was an significantly increase/decrease in inpartu pregnant women. Before the data samples were analyzed using t-test (one side/one-tailed) mentioned above, the data will be analyzed with the prerequisite parametric test data normality by using Normal Probability Plot test, the value of the ratio of inclination and kurtosis ratio. The decision criteria when the Normal Probability Plot observed values around a diagonal line (green) and no observed values are out of the red boundary lines and the value of the ratio of inclination and kurtosis ratio is between -2 and +2, then concluded that the data were normally distributed [**10**]. As for all the calculation used data analysis software tools GENSTAT Procedure Library Edition Release 16.1 Release PL24.1.

**Ethical clearance:** This research was equipped with a feasibility study of ethics approval from Research Ethics Committee, Faculty of Medicine, University of Brawijaya.

## Results

**Figure 1:**
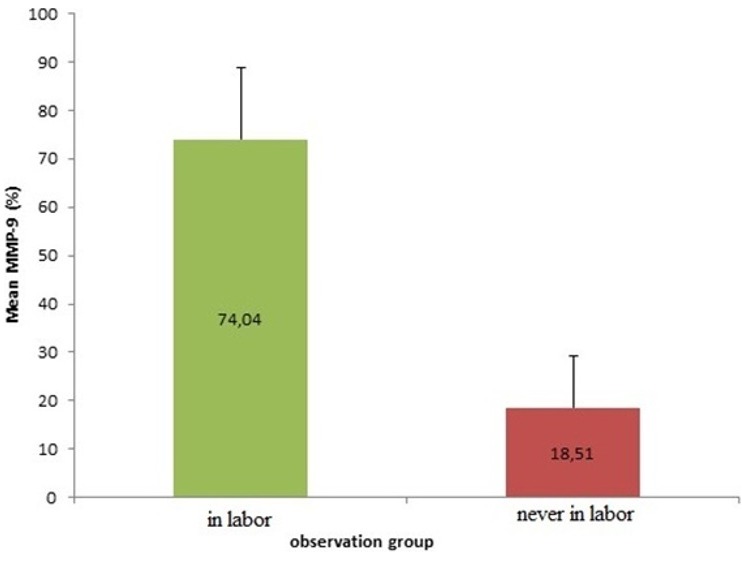
Comparison of MMP-9 expression of woman in labor compared to woman who is never in labor (significancy, p < 0.001)

**Figure 2:**
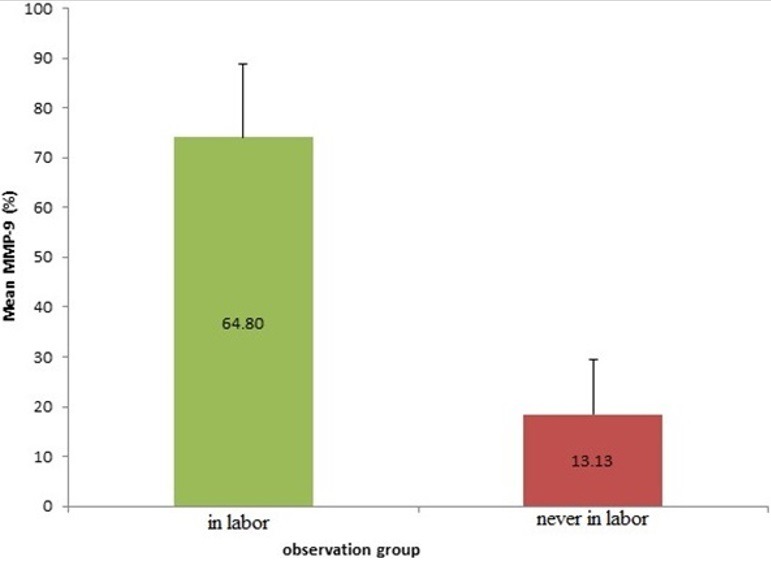
Comparison of MMP-2 expression of woman in labor compared to woman who is never in labor (significancy, p < 0.001)

**Figure 3:**
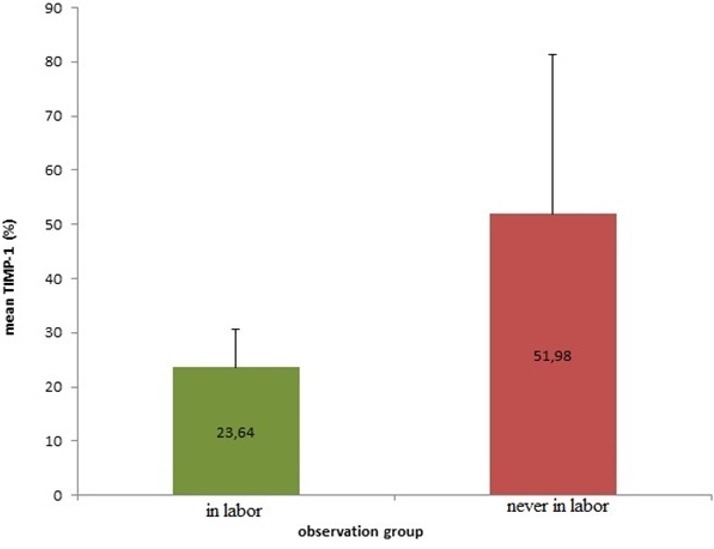
Comparison of TIMP-1 expression of woman in labor compared to woman who is never in labor (significancy, p < 0.001)

The result showed that there are significant differences mean levels of MMP-9 in the group of pregnant women in labor (74.04 ± 14.84%) with the group of pregnant women who are not in labor (18:51 ± 10.88%). It means that MMP-9 in the pregnant women in labor is much higher than the mean levels of MMP-9 of pregnant women who are not in labor (Figure 1). It can be concluded that there are increased levels of matrix metalloproteinase-9 (MMP-9) in the uterosacral ligament of inpartu pregnant women. The result showed that there are significant differences of mean levels of MMP-2 in the group of pregnant women in labor (64.80 ± 27.36%) with a group of pregnant women who are not in labor (13.13 ± 6.78%). The levels of MMP-2 in woman in labor is much higher than the levels of MMP-2 of woman who are not in labor (Figure 2). It means that there are increased levels of matrixmetalloproteinase-9 (MMP-2) in the uterosacral ligament of pregnant women in labor. The result showed that there are significant differences in the mean levels of TIMP-1 in pregnant women in labor group (23.64 ± 6.93%) with the pregnant women who are never in labor group(51.98 ± 29.45%). The TIMP-1 mean levels stem in the group of pregnant women in labor is much lower than the TIMP-1 mean levels stem of pregnant women who are not in labor group (Figure 3). It means that there is a decrease levels in TIMP-1 sacrouterine ligament of pregnant women in labor.

## Discussion

In the comparative test results mean levels of MMP-9, MMP-2 in the sacrouterine ligament of women in labor groups compared to women who are not in labor groups using independent samples t-test (independent sample t test) showed no statistically significant difference (p < 0.001). The presence of strain during childbirth which significantly affects the levels of Matrix Metalloproteinase-9 and MMP-2 are found in the extracellular matrix at uterosacral ligaments of pregnant women in labor. This process allows the baby's head stretches the stress strain of the pelvic floor tissue [11]. Stimuli strain which received by the extracellular matrix (MES) will be delivered to this .Stimuli fibroblast cells will through the role of integrins and fibronectin. This stimuli will give a signal to fibroblasts to differentiate and provide a signal transduction pathway to upregulation of MMP-9 and TIMP-1 [12]. MMP-9 thought to be related to cell migration, invasion and tissue remodeling in the reproductive process [13]. In the mouse model, the pregnancy effects of stiffness and relaxation significant vaginal tissue, stimulation distended vagina during delivery resulted in increased activity of MMP-2, MMP-9, and proteolytic enzymes that are responsible for the degradation and turnover of connective tissue in the vaginal wall of pregnant rats [14]. In vivo studies in humans found the intensity of MMP-9 in the myometrium during labor is stronger than before delivery. Prostaglandins enhance the ability of degradation of extracellular matrix by increasing MMP-2 and MMP9 and decreasing TIMP-1 through a positive feedback mechanism of the disintegration of the decidua during labor [15]. In the study, membrane amniotic fetus and humans have demonstrated an increase in the activity of MMP-9 during childbirth [14]. We have demonstrated a decrease in the activity of TIMP in amniotic fluid at birth, and at the same time we get an increase in MMP-9 on amniotic fluid and amniotic membranes of pregnant women at term gestation and preterm [13]. MMP-9 also plays a role in the involution of the uterus and cervical ripening. The study reported that MMP-9 also plays a role in preterm labor and cervical maturity [16, 17].

## Conclusion

There are increased levels of Matrix Metalloproteinase-9 and Matrix Metalloproteinase-2 with the decreased of TIMP-1 in the uterosacral ligament of primigravida women who underwent cesarean section because of obstructive labor at stage 1 and 2.

### What is known about this topic

- Pelvic organ prolapse (POP) or urogenital prolapse, is a decline in the pelvic organs that cause protrusion of the vagina, uterus or both;
- In vitro studies indicate that the cervical portion of the uterosacral ligament supporting over 17kg weight before decreasing function;
- The key element of the pelvic supporting system is the quantity of stability, ultra structure, and organization of extracellular matrix proteins such as collagen, fibronectin, and elastin, includes receptors such as integrins.

### What this study adds

- There are differences in the levels of Matrix Metalloproteinase-9, Matrix Metalloproteinase-2, and TIMP-1 in the uterosacral ligament of primigravida women in labor compared with women who are never in labor;
- The levels of Matrix Metalloproteinase-9 and Matrix Metalloproteinase-2 was increasing even the TIMP-1 in the uterosacral ligament of primigravida was decreasing; it caused by obstructive labor at stage 1 and 2 in women who underwent cesarean section;
- In vivo studies in humans found the intensity of MMP-9 in the myometrium during labor is stronger than before delivery; prostaglandins enhance the ability of degradation of extracellular matrix by increasing MMP-2 and MMP9 and decreasing TIMP-1 through a positive feedback mechanism of the disintegration of the decidua during labor.

## Competing interests

The author declare no competing interest.
